# An exploratory analysis of the relationship between ultraprocessed food consumption, alcohol intake, body composition, and cardiometabolic markers in individuals with alcohol use disorder

**DOI:** 10.1111/acer.70140

**Published:** 2025-08-13

**Authors:** Jennifer J. Barb, Lillian C. King, Shanna Yang, Sara Turner, Carlotta Vizioli, Ryan E. Tyler, Kong Y. Chen, Mehdi Farokhnia, Gwenyth R. Wallen, Lorenzo Leggio

**Affiliations:** ^1^ Translational Biobehavioral and Health Promotion Branch, Clinical Center National Institutes of Health Bethesda Maryland USA; ^2^ Nutrition Department, Clinical Center National Institutes of Health Bethesda Maryland USA; ^3^ Clinical Psychoneuroendocrinology and Neuropsychopharmacology Section, Translational Addiction Medicine Branch, National Institute on Drug Abuse Intramural Research Program and National Institute on Alcohol Abuse and Alcoholism Division of Intramural Clinical and Biological Research National Institutes of Health Baltimore Maryland USA; ^4^ Human Energy and Body Weight Regulation Core, National Institute of Diabetes and Digestive and Kidney Diseases National Institutes of Health Bethesda Maryland USA

**Keywords:** alcohol use disorder, diet quality, ultraprocessed food

## Abstract

**Background:**

Ultraprocessed foods (UPFs) are often high in salt, fat, and sugar and low in fiber and nutrients. Research has suggested that UPFs are associated with all‐cause mortality and have recently been proposed to align with properties of addictive substances. While research suggests that people with alcohol use disorder (AUD) have poor dietary habits in general, little is known about whether these people consume more UPFs than those without AUD. In an exploratory analysis, we examined how UPFs, diet quality, and added sugars consumption would be associated with other health outcomes in newly abstinent and currently drinking people with AUD and compared those to healthy individuals.

**Methods:**

Participants were individuals with AUD who were newly abstinent or currently drinking, and healthy controls. Two weeks of food intake records in an outpatient setting were assessed for UPF, diet quality, and added sugars, and were compared between the three groups. Correlations were assessed across diet and alcohol measures, body composition, resting energy expenditure, and atherosclerotic cardiovascular disease (ASCVD) risk scores within each group.

**Results:**

All groups consumed similar poor quality diets with >55% usual foods from UPFs and >8% of energy intake from added sugars. Within groups, only the newly abstinent individuals showed associations between ASCVD risk and alcohol use history along with diet quality and body composition.

**Conclusions:**

Despite the lack of statistically significant differences in diet quality measures between groups, nutrition support for healthier food choices in people with AUD, especially during alcohol recovery, should be carefully investigated.

## Graphical abstract

This preliminary work highlights the high consumption of ultraprocessed foods (UPFs) and poor diet quality among individuals with alcohol use disorder (AUD), regardless of drinking status, as well as in controls without AUD. While no significant group differences were found, the findings underscore the need to address dietary behaviors in general and in AUD treatment. Future larger longitudinal studies are needed to explore how diet influences long‐term outcomes in individuals with AUD.

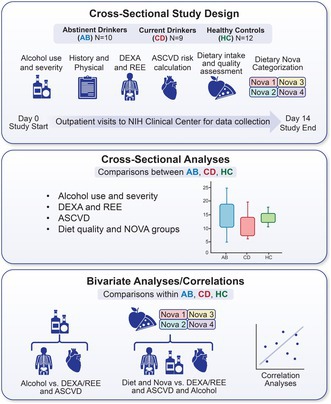

## INTRODUCTION

Alcohol use disorder (AUD) is a chronic medical disorder characterized by problematic alcohol use, leading to significant impairment and consequences across many aspects of the individual's life, including social, physical, mental, occupational, and legal domains (American Psychiatric Association, 2022; Westman et al., 2015). In 2023, approximately 28.9 million people aged 12 and older had AUD, representing 10.3% of the US population in that age group (SAMHSA, 2023). People with AUD often have other co‐existing medical and mental health disorders, including nutritional deficiencies (Castillo‐Carniglia et al., 2019; Manari et al., 2003; Ross et al., 2012; Wilkens Knudsen et al., 2014). Nutritional deficiencies in people who drink alcohol heavily are due to poor dietary habits and malabsorption of both macronutrients such as carbohydrates and micronutrients such as thiamine and other B vitamins (Butts et al., 2023; Vassallo et al., 2020). Additionally, individuals with AUD often derive a significant portion of their caloric intake from alcohol, displacing solid food (Bergheim et al., 2003; Jophlin et al., 2024). Despite the link between AUD and malnutrition, research investigating dietary intake behaviors and diet quality in people with AUD is sparse. For example, to the best of our knowledge, only one study has investigated diet quality using the Healthy Eating Index‐2015 (Reedy et al., 2018) in patients with AUD during an inpatient treatment program (Reedy et al., 2018; Yang et al., 2022); and only one other study assessed diet quality using the number of ultraprocessed foods (UPFs) consumed as a measure of diet quality, where the authors showed that people with AUD consumed twice as much UPFs than healthy controls and that insufficient dietary fiber intake was linked to AUD‐related malnutrition (Amadieu et al., 2021).

The assessment of UPF consumption in dietary intake studies has gained increasing attention in epidemiological research since the introduction of the Nova classification in 2009 (Monteiro, 2009), which categorizes foods into four groups based on their extent and purpose of processing, with UPFs falling into the Nova 4 category. Since then, there has been a large focus in nutritional research on the adverse health impacts of UPFs (Baker et al., 2020; Hall et al., 2019; Monteiro, 2009; Monteiro et al., 2019). A recent meta‐analysis of 45 pooled studies supported associations between greater UPF consumption and adverse health outcomes, for example, higher risks of cardiovascular disease (CVD)‐related mortality, type 2 diabetes mellitus, and anxiety and other mental health disorders (Lane et al., 2024). For example, each daily serving of UPFs was associated with a 9% increase in the risk of CVD mortality in the prospective Framingham Offspring Cohort (Juul et al., 2021). Alcohol is classified as a processed food (Nova 3) when it is produced through fermentation of unprocessed or minimally processed foods (Nova 1), such as with beer, cider, and wine. When alcohol is further refined through distillation following fermentation, it is categorized as an UPF (Nova 4). Distilled spirits such as whisky, gin, rum, and vodka are categorized as Nova 4. It is expected that people who heavily use alcohol would be deriving a large proportion of their calories from UPFs (Steele et al., 2023). Further complicating this, diets high in added sugars may exacerbate alcohol craving, potentially leading to increased risk of return to alcohol drinking in AUD, while also impacting mood regulation (Abrantes et al., 2022; Braun et al., 2021).

Interestingly, recent work proposes that UPFs may be addictive substances (Gearhardt & DiFeliceantonio, 2023) with some studies suggesting that “UPF addiction” is prevalent among individuals with Binge Eating Disorder and Bulimia Nervosa (Gearhardt & DiFeliceantonio, 2023; LaFata et al., 2024). Despite the link with poor health outcomes, around 60% of the standard American diet consists of UPFs, primarily from breads, soft drinks, and baked goods (Steele et al., 2023). Understanding how UPFs are consumed in people with addiction is critical, especially during recovery given the link between AUD and malnutrition (Di Nicola et al., 2015). An early study by Kampov‐Polevoy et al. (1997) found that abstinent individuals with AUD preferred higher levels of sugar in sucrose solutions compared to those without AUD, suggesting that people with AUD in recovery may choose more sugary types of foods, such as UPFs, which often contain high amounts of added sugars. There have been more recent studies further supporting this link of heightened sugar preferences in people with AUD (Bouhlal et al., 2018; Kampov‐Polevoy et al., 2003; Lange et al., 2010). This parallel suggests that addressing UPF consumption during AUD recovery could help prevent substituting one addictive behavior for another, thus enhancing treatment outcomes. However, while the “substitution theory” supports this idea, the literature still lacks studies specifically investigating UPF consumption in individuals with AUD, underscoring the need for further research on this potential link and its implications for addiction recovery.

The primary aim of this exploratory study was to better understand diet quality and UPF consumption in individuals with AUD, specifically those who are newly abstinent and those currently drinking. This question has not previously been assessed within this patient population. Given the limited sample size and potential for Type II error, our analysis focused on identifying trends and generating hypotheses for future research, rather than drawing definitive conclusions. To support this aim, we conducted a comprehensive assessment of dietary intake, including the proportion of energy derived from added sugar and UPFs, and evaluated overall diet quality using the Healthy Eating Index 2015. These metrics were compared to matched controls without AUD. In addition, we explored potential associations between dietary factors and clinical characteristics—including alcohol use history and severity, body composition, resting energy expenditure, and ASCVD risk—across the three groups. These analyses were intended to uncover preliminary patterns and relationships that warrant further investigation in larger, more powerful future studies.

## METHODS

### Study population and data collection

This study is a secondary and exploratory analysis from a cross‐sectional protocol (NCT03152760) investigating gut microbiome associations in individuals with AUD; the primary outcomes/analyses are reported elsewhere (Piacentino et al., 2024). The study ran from August 2017 to February 2020 and was terminated early due to the COVID‐19 pandemic and the closure of bringing in participants for clinical studies at the NIH. Thus, the planned sample size was not fully met.Participants were screened via the National Institute on Alcohol Abuse and Alcoholism (NIAAA) natural history protocol (NCT02231840), and eligible participants were then enrolled into this study after providing informed consent (see Supplemental Methods, for eligibility criteria in Data S1). The protocol and all related procedures (including participant compensation) were approved by the appropriate National Institutes of Health (NIH) Institutional Review Board.

**FIGURE 1:**
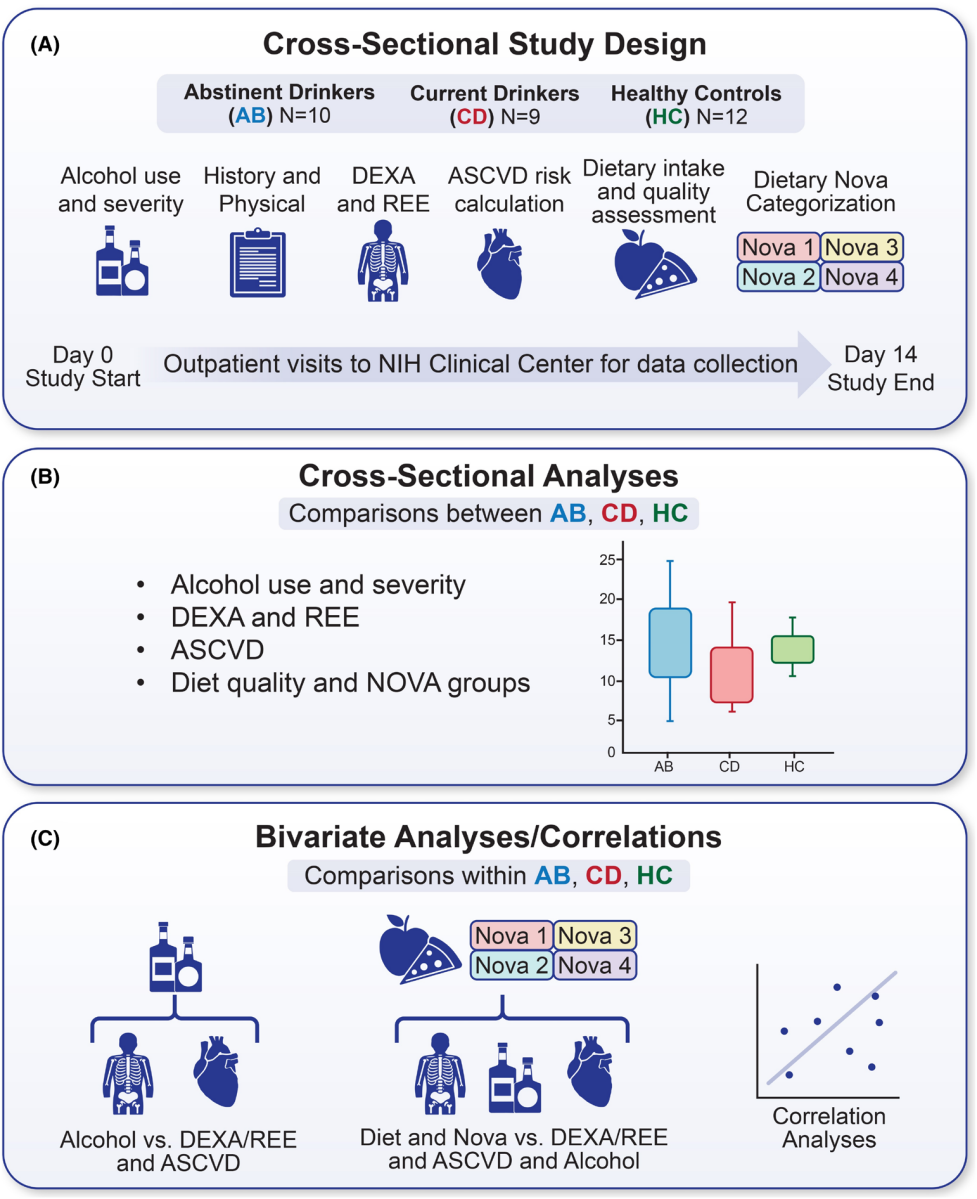
Study design and analytical workflow. Study design and analytical workflow. (A) Descriptive flow of data collection and study design during the 2‐week sampling window. (B) Cross‐sectional analyses across the three groups investigating differences in alcohol use/severity, body composition (DEXA) and resting energy expenditure (REE), cardiovascular disease risk (ASCVD), diet quality and Nova categorization. (C) Within group assessment using multivariate correlations of variables.

This study included three groups: treatment‐seeking, newly abstinent individuals with AUD (ABs, *N* = 10), nontreatment seeking, currently drinking individuals with AUD (CDs, *N* = 9), and healthy controls (HCs, *N* = 12). Data collection occurred during multiple outpatient visits at the NIH Clinical Center in Bethesda, MD, USA (Figure 1A). AUD and other potential mental health conditions were evaluated via the Structured Clinical Interview for the Diagnostic and Statistical Manual of Mental Disorders, Fifth Edition (DSM‐5, SCID‐5) (American Psychiatric Association, 2022; First et al., 2015). The AB group was treatment‐seeking and had recently completed inpatient treatment for approximately 4 weeks at the NIH Clinical Center and remained abstinent, following discharge, for a minimum of 2 weeks prior to enrollment in this study (hence at least 6 weeks of abstinence in total prior to enrollment). Both ABs and CDs met NIAAA criteria for heavy drinking at baseline. The HCs were BMI, age‐, and sex‐matched, did not meet criteria for current of past AUD diagnosis, and were low or nondrinkers (women ≤ 1 drink/day, men ≤ 2 drink/day) (U.S. Department of Health and Human Services, U.S. Department of Agriculture, 2020). Total cholesterol, high‐density lipoprotein (HDL), low‐density lipoprotein (LDL), triglyceride, and hemoglobin A1c (HbA1c) levels were assessed twice per participant through the study. For descriptive methods on body composition and resting energy expenditure collection, atherosclerotic cardiovascular disease risk score and social vulnerability index (SVI) calculations, please see Supplemental Methods for further details (Data S1).

### Alcohol‐related measures

The Diagnostic and Statistical Manual of Mental Disorders, 5th Edition (DSM‐5) provides specific criteria for diagnosing AUD and classifies its severity based on the number of criteria (American Psychiatric Association, 2022). There are 11 criteria, covering four main domains: Impaired control (e.g., drinking more or longer than intended, unsuccessful attempts to cut down); Social impairment (e.g., failure to fulfill roles at work/school/home, continued use despite social problems); Risky use (e.g., using alcohol in hazardous situations); and biological/pharmacological effects (tolerance and withdrawal). Severity is determined by how many criteria are met: Mild AUD: 2–3 criteria; Moderate AUD: 4–5 criteria; and Severe AUD: 6 or more criteria. To be diagnosed with AUD at all, the person must meet at least two of the 11 criteria within the same 12‐month period. Current diagnosis of AUD, that is, within the past 12 months, was an inclusion criterion for AB and CD groups.

The *Alcohol Use Disorder Identification Test* (AUDIT) is a 10‐item questionnaire assessing alcohol consumption, drinking behaviors, dependence, and alcohol‐related problems. Questions are scored 0–4, and the total score ranges from 0 to 40, with higher scores indicating more harmful drinking alcohol (Saunders et al., 1993). The *Alcohol Dependence Scale* (ADS) is a 25‐item questionnaire that measures the severity of alcohol dependence over the past 12 months. ADS scores range from 0 to 47, where higher scores reflect greater severity of alcohol use/dependence (Skinner & Horn, 1984). The Alcohol Timeline Follow Back (TLFB) is a structured interview assessing alcohol consumption, according to the number of standard drinks. One standard drink is defined as any alcohol‐containing beverage with at least 14 g of pure alcohol in the United States. For this study, TLFB was administered for the past 90 days (National Institute on Alcohol Abuse and Alcoholism (NIAAA), 2024; Sobell et al., 1979). The *Lifetime Drinking History* (LDH) examines alcohol use throughout the lifespan and is a retrospective interview‐based procedure that is used to identify patterns of alcohol use and abuse, which characterizes the timeframe from the start of regular drinking to the current drinking behavior (Koenig et al., 2009). A participant's number of years of heavy drinking is devised from this questionnaire.

In this study, the DSM‐5, AUDIT, ADS, TLFB, and LDH were used to assess alcohol use severity. For the AB group, the average drinks/day, the number of heavy drinking days, and the number of heavy drinking years captured in the time window prior to inpatient enrollment are used in the current analysis; for the CD and HC groups, these values are captured at the start of the parent study (NCT03152760). Days since the last alcohol‐containing drink were calculated for the AB group based on the time between the last drink, as reported on the 90‐day TLFB (prior to treatment) and the start of this study. To gather more detailed information on alcohol use, the history and physical intake, performed at the start of the protocol, were reviewed to obtain information about drink preference type (wine, beer, and liquor). Descriptive information about each participant's preferred alcohol type was extracted (Table S1).

### Dietary intake assessment

Dietary intake including food and alcohol was assessed using food records completed at home by participants throughout the study. Food records were collected and reviewed for accuracy and completeness by trained nutrition staff. All dietary intake data were collected and analyzed using the 2016–2019 Nutrition Data Systems for Research (NDSR) software (University of Minnesota, Minneapolis, MN). For this secondary analysis, provided food records were used to generate data reflecting nutrient intake, as well as measures of diet quality assessed by the Healthy Eating Index (HEI)‐2015 scores and for Nova classification (described below). The HEI‐2015 assesses how well the individual's diet aligns with key recommendations and dietary patterns published in the US Department of Agriculture 2015–2020 Dietary Guidelines for Americans (U.S. Department of Health and Human Services, U.S. Department of Agriculture, 2015). The steps used to calculate HEI‐2015 scores included (1) summing the intake of relevant nutrients or food groups used in each HEI component across days of reported intake per person; (2) constructing ratios for each person, including the ratio of dietary constituents per 1000 kcals, percent of energy or ratio of fatty acid intake; (3) scoring the ratios according to the HEI scoring standards for each component; and (4) calculating the mean component and total score for each group. The HEI‐2015 total score ranges from 0 to 100, with a higher score indicating better alignment with the Dietary Guidelines for Americans (Reedy et al., 2018). Dietary intake averages for daily dietary fiber (grams/day) and added sugars (grams/day), as well as the proportion of energy from carbohydrates, protein, fat and added sugars are reported. Additionally, total energy intake is reported, both including and exclusding emergy from alcohol.

### Nova classification methodology

All alcohol‐containing beverages consumed were excluded from the Nova classification procedure, following the approach of Amadieu et al.'s, 2021, to address the primary aim of this study: determining whether calories from UPFs differ across the groups. Accordingly, only food and nonalcoholic beverages were categorized into Nova groups. Prior to Nova classification, all unique foods consumed by participants were reviewed by dietitians and study team members for categorization into one of the following types: (1) single ingredient items or (2) mixed dish items. Mixed dish items were further categorized into of “industrial origin” or “culinary preparation,” in line with Martinez‐Steele et al., 2023, (Figure S1). Mixed dishes of culinary preparation were disaggregated into their single item ingredients. All items were then classified into their respective Nova group as follows: Nova 1: minimally processed foods (MPF) (i.e., whole food items such as fruits, vegetables, milk), Nova 2: culinary ingredients (i.e., flour, sugar, butter, oil), Nova 3: processed foods (PF) (i.e., cured meat, salted nuts), or Nova 4: UPFs. UPF are foods of industrial origin and are manufactured and sold as ready‐to‐eat foods with preservatives and ingredients not found in a home kitchen. For any single food item that was ambiguously named such as “unknown if salted,” an assumption of higher processing was made throughout this scoring procedure and the higher Nova classification was used. The total proportion of energy (excluding all alcohol intake) provided by all foods and nonalcohol beverages for each Nova classification group was calculated. Total energy for each Nova category was divided by the total overall intake for each person to obtain the proportion of energy per Nova group per person.

### Statistical analysis

JMP® version 16 Statistical Computing Software (SAS Headquarters, Cary, NC) was utilized for data visualization and statistical testing. Descriptive statistics (mean and standard deviation for continuous data, frequencies, and percentages for categorical data) were computed for all study variables for the three groups. Cross‐sectional analyses on demographics, alcohol, body composition, lipid biomarkers, dietary, DEXA, REE, and smoking variables between the three groups were tested using paired nonparametric testing procedures (Kruskal–Wallis test for continuous data and chi‐squared test for nominal data) (Figure 1B). Given the small sample sizes, we conducted preliminary tests to assess group differences in potential covariates. No significant differences were found (*p* > 0.05); thus, the need to control for covariates was not included in the final analyses. Nonparametric Spearman correlation analyses were conducted within each group between diet, DEXA, REE, ASCVD, and alcohol variables to assess within‐group associations (Figure 1C). To assess the impact of smoking on dietary intake, analyses were conducted separately in smoking and nonsmoking groups as well as between smoking and nonsmoking groups. Correlations of State and National SVIs with BMI, education years, and diet measures were assessed across the entire cohort using Spearman correlation analyses. To address limitations in statistical power and to provide information on the magnitude of group differences, we calculated effect sizes for Kruskal–Wallis tests using epsilon squared (*ε* ^2^). Epsilon squared values were interpreted using standard guidelines, with 0.01, 0.06, and 0.14 indicating small, medium, and large effect sizes, respectively. Effect sizes were reported for all Kruskal–Wallis tests regardless of statistical significance to highlight clinically meaningful group differences.

Multiple comparisons were corrected using a false discovery rate (FDR) <25% (Benjamini & Hochberg, 1995). A significance threshold was set at two‐tailed *p* < 0.05 and FDR <25%.

## RESULTS

### Sample characteristics

Participant demographics were similar across most metrics (*p* > 0.05) (Table 1). ABs smoked significantly more cigarettes per day (12.52 ± 7.47) than CDs, who smoked on average 2.99 ± 3.27 cigarettes per day (*p* < 0.001). Social determinants of health, including marital status and household income, were similar across groups except for the number of education years (*p* = 0.017), which was significantly higher among CDs (16.5 ± 2.9) compared to the ABs, who had the lowest (13.3 ± 2.5) (Table 1, Table S2). There were no significant differences in state and national SVIs among the groups (Table 1) nor were there any significant correlations between state/national SVIs and BMI, education years, drinking measures, ASCVD risk score, diet quality, proportion of energy from added sugars, or UPFs.

**TABLE 1:** Participant demographics and clinical diagnostic markers.

Characteristic, mean (SD) or *N* (%)	AB (*n* = 10)	CD (*n* = 9)	HC (*n* = 12)	Test	*ε* ^*2*^	*p*
Age (years)	45.9 (11.4)	45.0 (12.6)	48.8 (12.0)	*H* = 0.534	0.0	0.766
Marital status						
Divorced/Single/Widowed	7 (70%)	7 (77.8%)	6 (50%)	*χ* ^2^ = 1.92		0.389
Married	3 (30%)	2 (22.2%)	6 (50%)			
Education (years)	13.3 (2.5)	16.5 (2.9)	16.3 (2.5)	*H* = 8.11	0.169	**0.017** ^ac^
Annual household income ($)						
<29 K	3 (30%)	3 (33.3%)	3 (25%)	*χ* ^2^ = 1.863		0.761
30–59 K	6 (60%)	4 (44.4%)	5 (41.7%)			
>75 K	1 (10%)	2 (22.2%)	4 (33.3%)			
Social Vulnerability Index						
State	0.59 (0.23)	0.46 (0.21)	0.54 (0.26)	*H* = 1.31	0.0	0.519
National	0.54 (0.25)	0.40 (0.23)	0.49 (0.24)	*H* = 1.18	0.0	0.555
BMI (kg/m^2^)	26.7 (5.1)	25.7 (4.0)	28.9 (4.9)	*H* = 2.42	0.014	0.299
Waist circumference (cm)	93.9 (12.8)	90.4 (9.9)	96.8 (11.6)	*H* = 2.030	0.001	0.362
Sex						
Male	8 (80%)	7 (77.8%)	8 (66.7%)	*χ* ^2^ = 0.592		0.744
Female	2 (20%)	2 (22.2%)	4 (33.3%)			
Race						
Asian	1 (10%)	0 (0%)	0 (0%)	*χ* ^2^ = 3.074		0.545
White	6 (60%)	7 (77.8%)	7 (58.3%)			
Black	3 (30%)	2 (22.2%)	5 (41.7%)			
Ethnicity						
NH‐Latino	10 (100%)	9 (100%)	11 (91.7%)	*χ* ^2^ = 1.636		0.441
Hispanic	0 (0%)	0 (0%)	1 (8.3%)			
Smoking status						
Smoker	6 (60%)	4 (44.4%)	0 (0%)	*χ* ^2^ = 9.848		**0.007**
Nonsmoker	4 (40%)	5 (55.6%)	12 (100%)			
Cigarettes per day	12.52 (7.47)	2.99 (3.27)	0 (0)	*H* = 23.592	0.419	**<0.001**
Drinking measures						
Days Abstinent^†^	63.5 (32.49)	NA	NA	NA	NA	**NA**
Average drinks/day	16.9^‡^ (8.1)	5.4 (1.5)	1.3 (1.3)	*H* = 25.53	0.440	**<0.001** ^a,b c^
Number of heavy drinking days	70.9^‡^ (26.3)	37.8 (27.9)	0.1 (0.3)	*H* = 25.02	0.434	**<0.001** ^abc^
Number of heavy drinking years	17.7^‡^ (9.9)	12.9 (13.2)	0.8 (2.3)	*H* = 13.86	0.283	**<0.001** ^bc^
ADS	21.5 (9.2)	10.2 (6.1)	0.4 (1.0)	*H* = 25.19	0.436	**<0.001** ^abc^
AUDIT	27.8 (7.7)	16.3 (5.6)	2.0 (1.6)	*H* = 24.71	0.431	**<0.001** ^abc^
DSM‐5 AUD	8.8 (1.62)	5.2 (2.43)	0.0	*H* = 24.78	0.813	**<0.001**
Lipid panel						
Total cholesterol	183.45 (24.49)	188.89 (32.72)	174.88 (25.47)	*H* = 0.836	0.042	0.659
HDL	46.3 (11.3)	61.0 (12.3)	54.3 (12.1)	*H* = 7.870	0.210	**0.019**
LDL	114.4 (19.94)	105.4 (32.2)	99.3 (21.7)	*H* = 2.035	0.001	0.36
Triglyceride	114.5 (54.85)	113.0 (42.08)	105.9 (41.41)	*H* = 0.211	0.064	0.900
HbA1c (mmol/mol)	5.21 (0.60)	5.23 (0.24)	5.37 (0.33)	*H* = 0.475	0.0	0.789
ASCVD score	5.65 (5.08)	3.70 (3.53)	5.22 (5.24)	*H* = 1.1918	0.029	0.5511
Blood pressure (mm Hg)						
Systolic	117.5 (10.5)	127.2 (12.2)	128.3 (16.2)	*H* = 3.6298	0.058	0.1629
Diastolic	74.0 (5.8)	75.1 (10.0)	78.1 (10.0)	*H* = 1.2677	0.026	0.5305
ASCVD score *N* (%)						
Low (<5%)	6 (60%)	6 (66.7%)	7 (58.3%)	*χ* ^2^ = 0.6494		0.985
Borderline (5% to <7.5%)	2 (20%)	1 (11.1%)	2 (16.7%)			
Intermediate (7.5% to <20%)	2 (20%)	2 (22.2%)	3 (25%)			

*Note*: Significant Post‐Hoc group tests *p* < 0.05: ^a^CD vs. AB, ^b^CD vs. HC, ^c^HC vs. AB. Bold values indicate *p* < 0.05.

Abbreviations: ADS, Alcohol Dependence Scale; ASCVD, atherosclerotic cardiovascular disease; AUDIT, Alcohol Use Disorder Identification Test; BMI, Body Mass Index; DSM5, Diagnostic and Statistical Manual, 5th Edition; H, H, statistic from Kruskal‐Wallis test; HbA1c, Hemoglobin A1c; HDL, high‐density lipoprotein; LDL, low‐density lipoprotein; NH, not‐Hispanic.

^†^ Days abstinent is calculated at the time the participant started the inpatient treatment program to the time the participant enrolled in the current protocol.

^‡^ 90‐day alcohol TLFB is calculated prior to treatment for the AB group.

### Previous alcohol use and severity

At the start of this study, ABs had been abstinent about 64.25 ± 32 days, with a range of 44–153 days overall (Table 1, Figure S2). Alcohol preference indicated that 50% of ABs preferred liquor, while most of the CDs (77.8%) preferred beer/wine (Table S1). As previously reported (Piacentino et al., 2024), prior to inpatient treatment, the AB group had significantly higher alcohol use severity (AUDIT and ADS) and significantly more average drinks per day, more heavy drinking days, and more heavy drinking years compared to both the CDs and HCs (Table 1, Table S2). Based on the number of DSM‐5 criteria, all individuals in the AB group met criteria for severe AUD (8.8 ± 1.5). In contrast, the CD group showed a range of AUD severity (5.2 ± 2.1): mild (*n* = 3), moderate (*n* = 2), and severe (*n* = 4) (Table 1).

### Lipid biomarkers, atherosclerotic cardiovascular disease risk, and body composition

When lipid biomarkers were assessed across groups, only HDL was found to have a significant difference, with the CD group having the highest average level (61.0 ± 12.3) (*ε* ^2^: 0.210; *p* = 0.019) (Table 1). All other lipid biomarkers and HbA1c were similar across the groups (Table 1). Additionally, ASCVD risk was similar across the three groups (*p* > 0.05), and most of the participants fell within the “low” risk category. No differences in body composition and resting energy expenditure (REE) assessments were observed (*p* > 0.05) (Table S3), despite the HCs having higher average BMI, total body fat, and higher percentages of total fat, android, and gynoid fat as well as abdominal fat than the CDs and ABs. When the ratio of dietary intake kcal/day compared to REE (kcal/day) was assessed, CDs had the highest ratio of 1.48 ± 0.294; but this was not significantly different from the other two groups (*p* = 0.067).

### Diet quality, added sugars, and Nova group assessment

**FIGURE 2:**
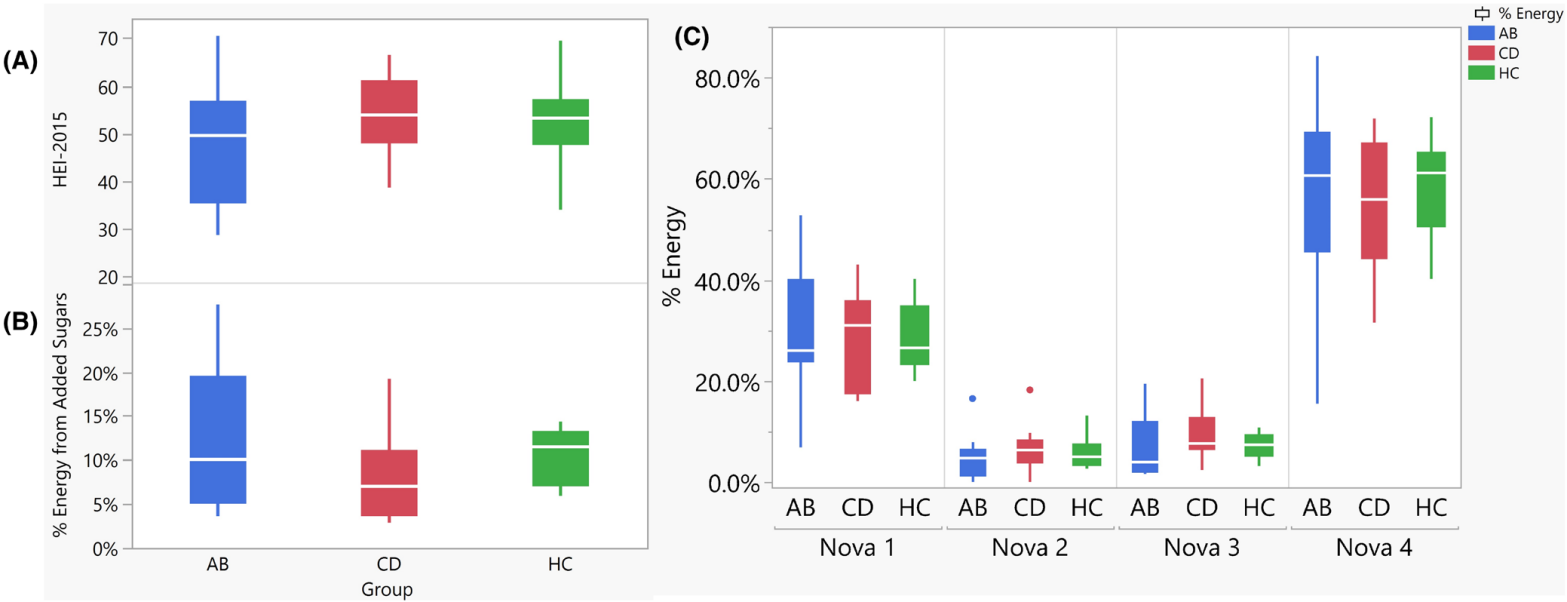
Diet quality, added sugars and proportion of energy from the four Nova categories across the three groups. Diet quality measured by (A) HEI‐2015 across the three groups is not significantly different (*p* = 0.522, *p* = 0.175, respectively). (B) Percent of energy from added sugars by total sugars (g) across the three groups is not significantly different (*p* = 0.395). (C) Percent of energy for each Nova category 1–4 across three groups (AB, CD, HC). No significant differences were observed within any of the four Nova categories across the three groups (*p* > 0.05). Participants consumed most of their energy from Nova 4: Ultraprocessed foods (UPFs).

Participants provided an average of 10 food record days throughout the study (Figure S3, Table S4). As previously reported, overall daily energy, fiber, added sugars, carbohydrate, and fat showed no significant differences (Piacentino et al., 2024). A marginal difference in energy from protein among the groups was observed (*p* = 0.049), but the effect size of this was 0.058, which approaches a moderate effect. As expected, alcohol energy (***ε*** ^***2***^ = 0.291, *p* < 0.001) was significantly different (Table S4). Diet quality, assessed by the Healthy Eating Index−2015 (HEI) (*p* = 0.522), and percent of energy from added sugars (*p* = 0.395), showed no significant difference across the groups (Figure 2A,B, Table 2). The effect of smoking on dietary intake measures was assessed since smoking status may influence appetite and taste preference. There were no significant differences observed across dietary intake measures within smokers nor within nonsmokers (Table S5). Additionally, no significant differences were observed between smokers and nonsmokers within the AB and CD groups (Table S6).

**TABLE 2:** Diet quality and percent of energy from Nova groups.

Dietary measure	AB (*n* = 10)	CD (*n* = 9)	HC (*n* = 12)	Test statistic	*ε* ^*2*^	*p*
Mean (SD)						
Diet quality						
Total HEI‐2015	47.9 (12.9)	54.2 (8.8)	51.6 (9.8)	*H* = 1.29	0.0	0.522
% Energy from added sugars	12.15% (8.5)	8.14% (5.31)	10.6% (3.05)	*H* = 1.85	0.0	0.395
Nova classification						
% kcal from						
Nova 1/MPF	30.70% (13.65%)	28.13% (9.81%)	28.63% (6.37%)	*H* = 0.220	0.0	0.896
Nova 2	4.88% (4.88%)	6.82% (5.08%)	5.98% (3.51%)	*H* = 1.58	0.0	0.459
Nova 3	7.11% (6.22%)	9.77% (5.30%)	7.29% (2.39%)	*H* = 2.38	0.013	0.304
Nova 4/UPF	57.30% (19.24%)	55.27% (13.49%)	58.10% (10.24%)	*H* = 0.282	0.0	0.869

*Note*: Kruskal‐Wallis tests.

Abbreviations: H, H statistic from Kruskal‐Wallis test; HEI‐2015, Healthy Eating Index‐2015; MPF, minimally processed foods; UPF, ultraprocessed foods.

**FIGURE 3:**
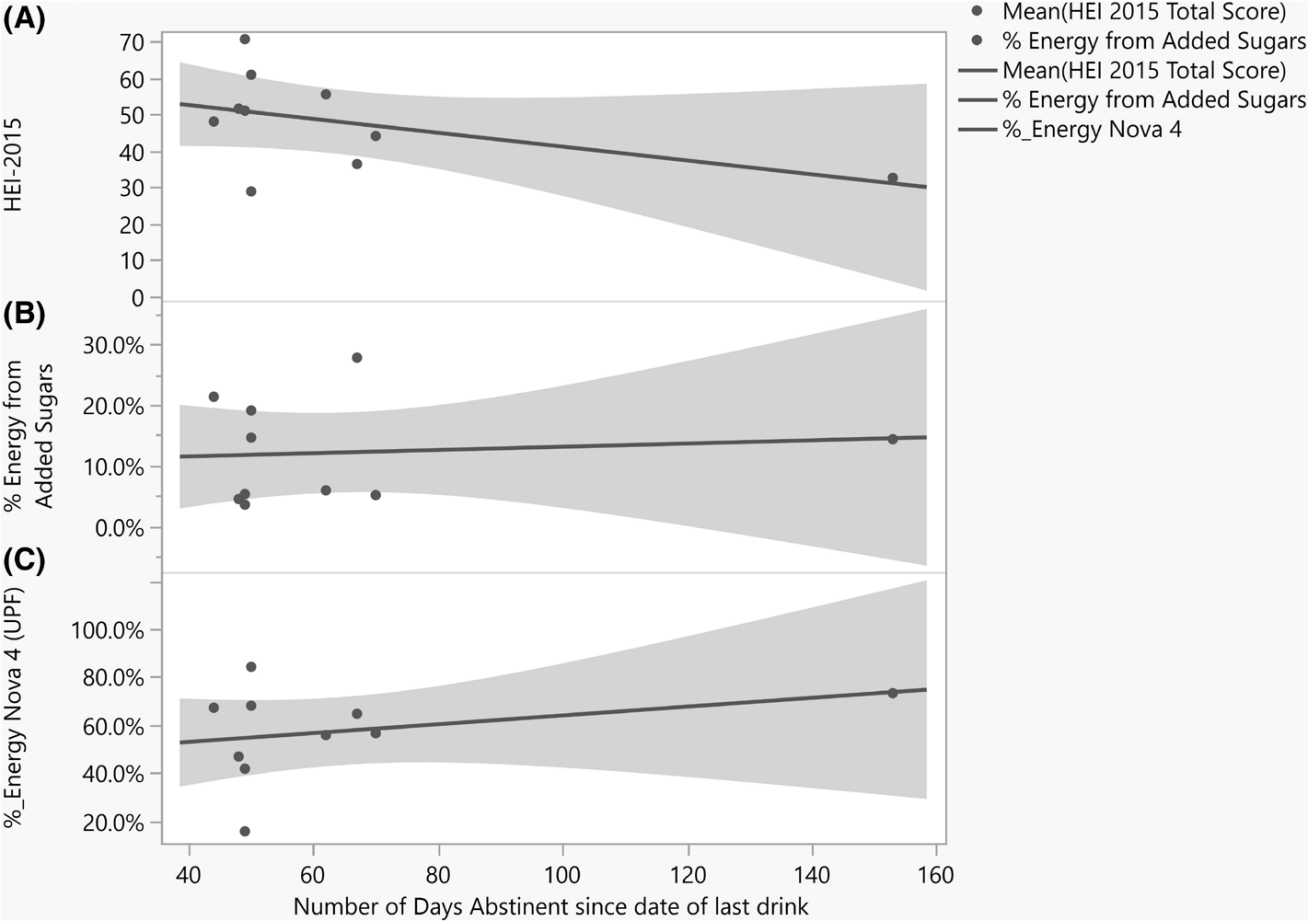
Association between number of abstinent days with dietary intake measures within AB group. Bivariate associations with dietary intake measures (*y*‐axis). Diet quality (A), percent of energy from added sugars (B) and (C) percent of energy from UPFs with number of days abstinent (*x*‐axis) within Abstinent individuals.

After the food categorization procedures (Supplemental Methods in Data S1) were completed and all foods and nonalcohol beverages were categorized into Nova processing groups, there were a total of 1511 unique food and beverages assessed and categorized (from 7412 total items, across all participants, all days, including repeatedly consumed items). Overall, ABs consumed 57.3% of their calories from UPFs, CDs consumed 55.3% of their calories from UPFs, and HCs consumed 58.1% of their calories from UPFs. No differences were found across the three groups for any of the four Nova categories (*p* > 0.05) (Table 2, Figure 2C). Within the AB group, the relationship between the number of abstinent days and diet measures was assessed, and no significant associations were observed with overall dietary quality (Figure 3A), (Spearman *ρ* = −0.445 *p* = 0.197), percent of energy from added sugar (Figure 3B), (Spearman *ρ* = 0.152, *p* = 0.674), or percent of energy from UPFs (Spearman *ρ* = 0.378, *p* = 0.281) (Figure 3C).

### Exploratory associations with diet, body composition, resting energy expenditure, cardiovascular disease risk, and alcohol measures

Multivariate correlation analysis was assessed within each group across all measures (Figure 1C); the Spearman correlation coefficients are plotted in a heatmap (Figure S4). There were five significant associations (*p* < 0.05) between drinking measures and body composition and REE within ABs and CDs, but the significance did not survive the multiple comparisons correction (FDR < 25%) (Figure S5).

**FIGURE 4:**
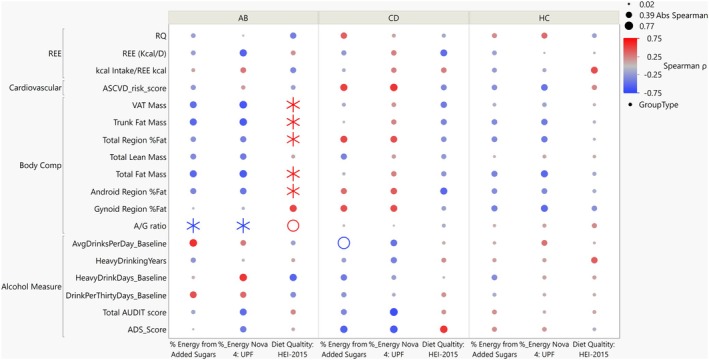
Significance correlation dot map between diet and REE, body composition, cardiovascular risk, and alcohol history across the three groups. Spearman correlation dot map showing associations with body composition, REE, ASCVD risk, alcohol severity and intake and diet quality, added sugar intake and processed and ultraprocessed food consumption. Color of the dots indicate direction of Spearman correlation coefficient. Size of the dot indicates absolute Spearman coefficient. Open circles “O” indicates *p* < 0.05; asterisk *Indicates FDR < 25%.

When dietary intake was assessed within groups and compared to alcohol use, body composition, REE, and ASCVD, there were seven significant associations observed within the ABs only after multiple comparisons correction (FDR < 25%) (Table 3); none observed within CDs or HCs. Two significant negative correlations between the A/G ratio and the % of energy from added sugars (Spearman *ρ* = −0.733, *p* = 0.016, FDR = 17.06%) and % energy of UPFs (Spearman *ρ* = −0.757, *p* = 0.011, FDR = 17.06%) were observed within ABs. Additionally, diet quality, measured by HEI‐2015, was positively associated with five of the body composition variables within ABs: % fat in android region (Spearman *ρ* = 0.685, *p* = 0.013, FDR = 22.28%), total fat mass (Spearman *ρ* = 0.733, *p* = 0.016, FDR = 17.06%), % total region fat (Spearman *ρ* = 0.685, *p* = 0.013, FDR = 22.28%), trunk fat mass (Spearman *ρ* = 0.733, *p* = 0.016, FDR = 17.06%), and Visceral Adipose Tissue (VAT) mass (Spearman *ρ* = 0.769, *p* = 0.009, FDR = 17.06%) (Table 3, Figure 4, Figure S6).

**TABLE 3:** Spearman correlations within AB and CD groups across diet, body composition, resting energy expenditure, cardiovascular disease risk, and alcohol measures.

	Variable	By variable	Spearman	*p*‐value	FDR (%)
Abstinent drinkers	% Energy from added sugars	A/G ratio	−0.733	0.016*	**17.06**
	% Energy Nova 4		−0.758	0.011*	**17.06**
	HEI‐2015	Android region %fat	0.685	0.029*	**22.28**
		Total fat mass	0.733	0.016*	**17.06**
		Total region %fat	0.685	0.029*	**22.28**
		Trunk fat mass	0.733	0.016*	**17.06**
		VAT Mass	0.769	0.009*	**17.06**
		A/G ratio	0.636	0.047	32.33
	ASCVD risk	Heavy drinking years	0.697	0.025	73.5
	Energy/REE kcal	Total AUDIT Score	−0.738	0.015	73.5
	vat mass	Total AUDIT Score	0.622	0.055	73.5
Current drinkers	% Energy from added sugars	Average drinks/Day	−0.683	0.042	83.4
	% Energy Nova 4	Total AUDIT Score	−0.655	0.055	83.4
	A/G ratio	Heavy drinking years	0.695	0.037	99.9
	ASCVD risk	Total AUDIT Score	−0.764	0.016	99.9

*Note*: Bold FDR indicates passed FDR < 25%.

* Indicates association passed FDR < 25%.

## DISCUSSION

This analysis examined the dietary habits, quality, and UPF consumption of currently drinking and recently abstinent individuals with AUD and compared those indices to healthy controls. We also evaluated the link between dietary measures and alcohol use history and severity, body composition, resting energy expenditure, and atherosclerotic risk across the three groups. Dietary intake measures assessed include percent of energy from UPF and added sugars as well as an assessment of diet quality using the Healthy Eating Index‐2015 (HEI‐2015). The three groups consumed a similar quality of diets as well as similar nonalcohol energy from UPFs and added sugars.

As previously reported (Piacentino et al., 2024), the three groups had similar total energy intake (excluding alcohol), as well as comparable macronutrient distributions, with 43.0% of energy derived from carbohydrates, 16.8% from protein, 35.6% from fat, and 10.3% from added sugars. Additionally, the average intake of total dietary fiber was 17.7 g, which is below the adequate intake levels recommended in the Dietary Reference Intakes (at least 21–26 g for adult females and 30–38 g for adult males) (U.S. Department of Health and Human Services, U.S. Department of Agriculture, 2015). When the proportion of energy from added sugars was assessed, there were no statistical differences between groups, and the participants consumed on average 10.3% of their daily energy from added sugars, though the range of intake for AB and CD was wider than HC (Figure 2A). The Dietary Guidelines for Americans (DGA) 2020–2025 recommends limiting added sugar to <10% of energy (U.S. Department of Health and Human Services, U.S. Department of Agriculture, 2015). Furthermore, all groups had poor alignment with the DGA recommendations, as indicated by low HEI‐2015 scores of on average 51.2. When using a graded approach to interpret these scores, all groups would receive a “grade of F” (Krebs‐Smith et al., 2018). Poor HEI‐2015 scores, combined with poor intake of dietary fiber, indicate suboptimal dietary behaviors across all groups, which may contribute to various health risks such as obesity, cardiovascular, and cardiometabolic diseases (Ahmad et al., 2020; Wu et al., 2015). Although our healthy control group did not meet key dietary targets outlined in the DGA (e.g., excessive intake of UPFs and added sugars, low fiber, and suboptimal HEI scores), their intake is reflective of broader national trends, as recent estimates suggest that UPFs account for more than 60% of energy intake among US adults. This suggests a ceiling effect and underscores the challenge of detecting significant differences between groups when baseline diet quality is already poor across the population. Furthermore, this represents a strength of our study, as it makes the results more generalizable, despite the small sample. The lack of significant differences in dietary intake indices between the groups may have been driven by small sample size. It is also possible that, since each group was slightly overweight, the similarly poor dietary behaviors may reflect comparable weight status rather than differences in drinking status. In other words, given the selection and matching criteria, all enrolled participants had suboptimal dietary habits, hence poor HEI‐2015 scores; therefore, drinking status did not lead to additional differences in diet and HEI‐2015 (potential ceiling effect).

Despite diet quality differences not reaching statistical significance, the AB group had the lowest diet quality (HEI‐2015) score (47.9) compared to HCs (51.6) and CDs (54.2). Consistent with previous literature, the treatment‐seeking AB group had more severe AUD, based on the number of DSM5 criteria and other measures, and reported greater comorbidities, which may have contributed to poorer over all diet quality (Haass‐Koffler et al., 2020; Lee et al., 2019; Ray et al., 2017; Rohn et al., 2017). Additionally, it has been previously shown that individuals with severe AUD may consume diets higher in processed foods and lower in fruits and berries (Amadieu et al., 2021; Rintamäki et al., 2014). Socioeconomically, ABs had fewer years of education (13.3) compared to CDs (16.5), which may also be a contributing factor, whereas previous research has shown a correlation between better diet quality and social determinants, such as education (McCullough et al., 2022). A novelty of this work is the inclusion of the Social Vulnerability Index (SVI), which is a measure to assess geographic vulnerability. Higher SVI scores represent greater area vulnerability, which has been associated with both increased food insecurity and higher bodyweight measures (An & Xiang, 2015; Ramphul et al., 2023). While three groups in this study showed nonsignificant differences in their national and state SVI scores, it is noteworthy that the AB group had the highest average SVI, indicating greater vulnerability.

Like diet, body composition and REE indices and ASCVD risk scores were similar across groups. Previous research on REE in people with AUD is mixed, with some studies showing higher REE in AUD individuals, while others report no effect, aligning with the current findings (Addolorato et al., 1997; Wagnerberger et al., 2007). Percentages of body adiposity were higher in the HCs than in the other two groups, which also is reflected by the BMI of the HCs. Overall, the participants in AB and CD groups appeared to have normal body composition and REE measures despite the AUD diagnosis. Additionally, the ABs showed a positive significant correlation with ASCVD risk and baseline heavy drinking days and average drinks per day. This finding is corroborated with previous research linking AUD with higher cardiovascular risk (Biddinger et al., 2022; Day & Rudd, 2019).

This study provides novel insights by presenting new data on UPF consumption in people with AUD, specifically newly abstinent individuals and currently drinking individuals with AUD, and people without AUD. Overall, the participants consumed 57% of their energy from UPFs, with no significant differences in energy consumed from UPFs across the three groups. This trend is similar to the typical American diet, which has been shown to have a large proportion of UPFs (about 57%) (Juul et al., 2018). These findings suggest that, within this small sample, individuals with AUD, whether currently drinking or in early recovery, consume diets similar to those of the general US population, particularly in terms of UPF intake. The absence of group differences may reflect limited statistical power due to the small sample size, increasing the likelihood of a Type II error. However, it is also plausible that the intentional matching of groups on key metabolic variables contributed to the observed similarities in dietary intake, as previously discussed. To the best of our knowledge, there has been only one other study by Amadieu et al. investigating UPF consumption in people with AUD, which showed that the participants with AUD consumed nearly twice the amount of UPFs than the healthy individuals. Although Amadieu et al.'s findings are not consistent with ours, there are inherent differences between the two studies that warrant some discussion. First, Amadieu, et al.'s study was conducted in Belgium, where the food choices are likely very different compared to those of the United States, where a large proportion of the American diet is simply comprised of UPFs. Furthermore, the type of dietary assessment performed in that study was a 3‐day recall, and the authors mention that the work may be subject to recall bias. The current study used food records completed at home daily by all participants, so the recall bias may have been alleviated by this method. Finally, Amadieu, et al.'s study looked at the number of UPFs consumed over the total number of foods consumed, whereas our current work investigated the energy contribution proportion from UPFs.

In this cross‐sectional analysis, we did not observe a significant association between length of abstinence and diet quality, percent energy from added sugars, or intake of UPFs among abstinent individuals. However, due to the cross‐sectional study design and limited power, we cannot infer temporal changes in dietary behaviors related to alcohol abstinence. Not only were variables assessed across the three groups, but relationships between dietary intake measures and body composition, resting energy expenditure, ASCVD risk, and previous alcohol use and severity were evaluated within each group. Overall, the only significant associations that passed very liberal multiple correction comparison filtering were observed in the recently abstinent individuals with a heavy alcohol use history, where diet quality (HEI‐2015) was significantly associated with five body composition measures. Interestingly, in the abstinent group (AB), higher HEI‐2015 scores were positively correlated with greater fat mass. One possible explanation is that diet quality was assessed during a period of abstinence, whereas fat mass may reflect the cumulative effects of prior heavy alcohol use and associated poor dietary behaviors. Thus, the observed association may not indicate that better diet quality leads to increased fat mass, but rather that improvements in dietary intake during abstinence have not yet resulted in measurable changes in body composition; the latter may require more prolonged abstinence before emerging. These findings highlight the importance of considering temporal factors when interpreting diet–body composition relationships, particularly in populations undergoing behavioral change. Additionally, the proportion of energy from added sugars and UPFs was negatively correlated with the A/G ratio, which is a measure of fat distribution that compares fat stored in the abdominal region to fat stored in the hip/thigh region. Although interpreting this finding is challenging, we speculate that higher consumption of added sugars and UPFs might promote greater abdominal fat accumulation relative to fat in the lower body. This could be of particular concern in individuals with a history of heavy alcohol use, as they may already be more prone to metabolic and cardiovascular diseases (Farinelli et al., 2021; Mirijello et al., 2017), as well as to increased abdominal fat deposition, which in turn is also linked to a higher risk of metabolic and cardiovascular issues. These findings underscore the importance of considering not just quantity but also the pattern of alcohol consumption when evaluating metabolic outcomes, particularly in populations with a history of alcohol use disorder.

After multiple comparisons correction, no other significant associations were observed. However, an inverse relationship emerged within the CD group between added sugar intake, alcohol consumption, and Nova 4 and AUDIT scores. While these correlations did not survive FDR correction, their effect sizes were moderate. These findings are in line with the food–alcohol competition theory (Cummings et al., 2017), which posits that individuals may substitute between food and alcohol as competing sources of reward. This is particularly relevant in populations prone to dietary restraint or disinhibition, where alcohol may suppress intake of highly palatable foods and vice versa. Though possibly driven by an outlier with very high alcohol use, this preliminary pattern merits further investigation in larger samples.

This study involved a thorough review of daily food records from all participants, which allowed for the examination of real‐life habitual diets in both individuals who continued heavy alcohol consumption and those who were recently abstinent. However, there are limitations of this exploratory study that should be discussed. As a secondary analysis, some foods were classified using the Nova categorization based on available information, without knowing exact brands or ingredients. Although dietary intake data were collected across all days of the week, the number of days varied by participant. We averaged intake across recorded days to minimize this variability, but differences between weekday and weekend eating patterns may still have introduced some residual bias. Additionally, potential recall bias cannot be ruled out. While BMI at screening was used to match groups in this study, it is important to note that BMI may not fully capture differences in body composition. Differences in measured waist circumference, body fat mass, and body fat percentage were detected, which were not accounted for in the initial matching process. Although no significant group differences in these measures were found, these variations may still influence metabolic outcomes. Therefore, we acknowledge that the groups were not entirely comparable in terms of body composition parameters, and this should be considered when interpreting the findings and in future studies. Finally, smoking status was not considered when matching the control group to the AB and CD groups, limiting the usefulness of the ASCVD risk scores and potentially impacting the food choices made by each group, as smoking impacts both appetite and taste; however, we conducted within‐group smoking differences and found no statistical differences between smokers and nonsmokers on diet measures. Most notably, the small sample size and early termination due to the COVID‐19 pandemic further limit the findings. The lack of statistically significant differences across some of the dietary intake indices assessed should be interpreted with caution, as this study was not fully powered. The results of this study are primarily hypothesis‐generating, and larger, longitudinal perspective studies are needed to better understand the relationship between early alcohol abstinence and changes in diet and UPF consumption in people with AUD.

In conclusion, this exploratory analysis provides valuable insights into dietary habits, UPF consumption, and their potential impact on individuals with AUD, both newly abstinent and those actively drinking. Despite no significant differences in dietary intake or UPF consumption across the groups, the study represents an interesting idea for future research to consider the role that diet quality and UPF consumption have on human health behaviors and outcomes, including individuals with AUD during recovery. UPFs are associated with several adverse health outcomes, including inflammation, mood disorders, and food addiction, all of which may complicate treatment and recovery from AUD (Gearhardt & DiFeliceantonio, 2023; Godos et al., 2023; Lane et al., 2024). For individuals recovering from AUD, high UPF consumption could exacerbate existing vulnerabilities, such as cardiovascular risk, metabolic dysfunction, and poor mental health, potentially increasing the risk of relapse and impeding progress in recovery. Given these risks, incorporating nutrition‐focused interventions that address UPF consumption and promote healthier food choices could be a key component of recovery plans. These findings suggest that early abstinence does not necessarily lead to immediate improvements in dietary behaviors, highlighting the need for further research and clinical strategies that integrate diet and nutrition as central components of AUD treatment. Future studies with larger, longitudinal designs are essential to better understand how diet, including UPF intake, influences long‐term recovery and return to drinking in people with AUD. Additionally, future research should explore specific UPF subtypes, such as sugar‐sweetened beverages and processed snack foods, to determine whether particular categories of UPFs are more strongly associated with negative outcomes in individuals with AUD.

## AUTHOR CONTRIBUTIONS

JJB, LL comprised the idea for the research topic. JJB and LCK analyzed the data. CV, RET, MF, and LL assisted with the management, analysis, and interpretation of the data. KYC oversaw the collection of the DEXA/REE data and assisted with the management, analysis, and interpretation of the DEXA/REE data. JJB, LCK, SY, ST provided all Nova food group categorization and assessed all dietary‐related variables. SY and ST assessed all dietary intake and provided food and dietary intake tables from the NDSR software. GRW and LL oversaw the workflow of the project. JJB and LCK wrote the first draft of the manuscript. All authors contributed to the final manuscript writing, editing, and review of the manuscript and approved its final version.

## FUNDING INFORMATION

This work was funded by National Institutes of Health Intramural Research Program; the ZIA‐DA000635 (Clinical Psychoneuroendocrinology and Neuropsychopharmacology Section; PI: Dr. Lorenzo Leggio), jointly supported by the NIDA Intramural Research Program (IRP) and the NIAAA Division of Intramural Clinical and Biological Research (DICBR); the Peter G. Dodge Foundation (PGDF) funding (Exploring Gut‐Brain and Brain‐Gut Interactions in Alcohol Use Disorder via Microbiota Investigations: A Pilot Study; PI: Dr. Lorenzo Leggio); the 1FI2GM154714–01: Postdoctoral Research Associate Training Program (PRAT) Fellowship (PI: Dr. Ryan E. Tyler), supported by the National Institute of General Medical Sciences (NIGMS) and the Center on Compulsive Behavior Fellowship, NIH (Fellow: Dr. Ryan E. Tyler). The contributions of the NIH authors were made as part of their official duties as NIH federal employees, are in compliance with agency policy requirements, and are considered Works of the United States Government. However, the findings and conclusions presented in this paper are those of the author(s) and do not necessarily reflect the views of the NIH or the U.S. Department of Health and Human Services.

## CONFLICT OF INTEREST STATEMENT

The authors have nothing to declare.

## Supporting information


Data S1

## Data Availability

All raw data are available upon request. To protect subject privacy, access to the clinical data is controlled by the Principal Investigator of the protocol (LL) and can be requested with a data sharing agreement.
