# Supplementary material for: An exploratory analysis of the relationship between ultraprocessed food consumption, alcohol intake, body composition, and cardiometabolic markers in individuals with alcohol use disorder
Source: Alcohol Clin Exp Res (Hoboken). 2025 Aug 13;49(10):2184–98. doi: 10.1111/acer.70140 (PMC12519058; doi:10.1111/acer.70140)
Supplement: Supplementary file 1 — Data S1 [file ACER-49-2184-s001.docx]

**Supplementary Material**

**Supplemental Methods:**

***Study Inclusion Criteria***

**All Participants**- Male or female individuals 21-70 years old (inclusive)

**Specific for Abstinent Group: AB**- Current Alcohol Use Disorder (AUD) by DSM-5 criteria
- Being alcohol abstinent for at least 4 weeks with minimum of 2 weeks in a non-protective environment at the time of study screening

**Specific for Current Drinking: CD**- Current Alcohol Use Disorder (AUD) by DSM-5 criteria
- Non-treatment seeking for alcohol use
-Satisfying heavy drinking criteria during 4-weeks prior to screening “for men, >14 standard drinks in any one week and ≥4 drinks per occasion at least once per month over the past 30 days; for women, >7 drinks per week and ≥3 drinks per occasion at least once per month over the past 30 days” and any drinking during the 2-day prior to signing the study-specific consent.

**Specific for Healthy Controls: HC**- No current or past diagnostic of AUD by DSM-5 criteria
- Non-alcohol drinkers or moderate alcohol drinkers i.e., up to 1 drink per day on average and not meeting NIAAA criteria for:
 - heavy drinking (i.e., for men, >14 standard drinks in any one week or ≥4 drinks per occasion at least once per month over the past 30 days; for women, >7 drinks per week or ≥3 drinks per occasion at least at least once per month over the past 30 days)
 - or binge drinking (i.e., drinking 5 or more standard drinks on the same occasion on at least 1 day in the past 30 days for both male and female)

***Study Exclusion Criteria***
- Current pregnancy or lactation.
- Positive urine drug test for illegal drugs.
- Presence of active implantable electronic devices (e.g., defibrillators, pumps, pacemakers).
- The following current medical conditions: diabetes; chronic gut inflammatory diseases; gastrointestinal or any other type of cancer; short bowel syndrome; conditions requiring parenteral nutrition.
- Self-reported diarrhea or other symptoms of possible enteritis (past 7 days).
- Recent history of sigmoidoscopy or colonoscopy (past 30 days).
- Current use (past 90 days) of the following medications: oral and/or intravenous (IV) antimicrobials (specifically: antiviral, antifungal, or antibiotics); prebiotics; probiotics; laxatives; antispasmodic drugs; oral, intramuscular (IM) or IV steroids
- Any other reason or clinical condition that the Principal Investigator (PI) or Medical Advisory Investigator (MAI) considers unsafe for the individual or not in the best interest of the study research integrity.

***Body composition and resting energy expenditure***Body composition was assessed using a dual energy x-ray absorptiometry (DEXA) scanner (GE Lunar iDXA, GE Healthcare, Madison, WI; software GE encore 15) and resting energy expenditure (REE) by indirect calorimetry (ventilated hood method, Parvomedic TrueOne 2400, Sandy, UT) one time during the study. Both DEXA and REE measurements were conducted at the National Institutes of Health (NIH) Clinical Center Metabolic Clinical Research Unit after ~12-hour fast. The following DEXA measures were assessed for group differences: total body and trunk fat, lean mass, visceral adipose tissue, total percent fat, percent android fat, percent gynoid fat, and the android/gynoid ratio. The gynoid region is the area around the hips and thighs, or the lower half of the body, android fat is the fat around the trunk and upper body, and the android/gynoid ratio is the ratio of the two. Along with all these measurements, participants Z-Score and T-scores were assessed. The REE measures included for this analysis for all participants were as follows: REE (kcal/day) and the ratio between total daily energy intake and REE (Energy (kcal)/REE), and the respiratory quotient (RQ) calculated as the ratio of carbon dioxide production to oxygen consumption.

***Atherosclerotic cardiovascular disease risk score and reported lipid data***
The atherosclerotic cardiovascular disease (ASCVD) risk score was calculated for all participants following the American College of Cardiology/ American Heart Association 2013 risk score guidelines (Goff Jr et al., 2014). The score is based on participant age, race, sex, smoking status, diabetes history, systolic blood pressure, blood pressure medication status, HDL, and total cholesterol. The score was calculated using the RCran project package ‘Cvrisk’ (Castro, 2023; R Core Team, 2023). All but one participant had two lipid panels during the study window, and the averages are reported for all participants having two scores. ASCVD scores were assessed to determine risk using the following categorization procedure: Low: 0 to 4.9, Borderline: 5 to 7.4, Intermediate: 7.5 to 20 and High: >20 (Arnett et al., 2019). One HC participant had a total cholesterol of 120, which is below the minimum possible total cholesterol of 130 that the R package can calculate, so an online calculator was used to find their ASCVD risk score (MDcalc, n.d.).

***Social Vulnerability Index (SVI) assessment***
This secondary analysis included an assessment of the social vulnerability index (SVI), which is a measure based on 15 social factors including demographic and socioeconomic factors (such as poverty, lack of access to transportation, and crowded housing) that adversely affect communities that encounter hazards and other community-level stressors. These values were achieved as follows: participants addresses were looked up on https://svi.cdc.gov/map/ and census tract numbers and national SVI scores were recorded from the map. State SVI information was looked up within the state specific information from censustract.gov. Each census tracts SVI score is calculated from 16 census variables falling into four broad categories: socioeconomic status, household characteristics, race and/or ethnicity, and housing type and transportation. SVI scores have an inverse relationship meaning that higher values indicate more vulnerable areas and lower scores indicate less vulnerable areas, and they range from zero to one (Flanagan et al., 2011). The SVI metrics included are reported for all but two participants because one was not a US resident, and one reported a PO box address.

***Culinary and Industrial detailed classification methods***Individual foods consumed by all study participants were reviewed and categorized into one of the following types: 1) single ingredient items (SII), or 2) mixed dish item, which was further categorized to be from 2a) industrial origin (Ind) or 2b) from culinary preparation (Cul) (see also **Supplemental Figure S1A**). Details from the food records such as location of meal, name of restaurant, or other hand-annotated details were referenced when determining if a mixed dish was from industrial or culinary preparation. Foods deemed as mixed dish- culinary preparation (2b) were disaggregated into their single food ingredients.

1. S*ingle ingredient item (SII):* Either a whole food (i.e., apple, potato, steak, broccoli), or a whole ingredient such as flour or butter.
2. *Mixed Dish:
   a. Industrial:* Packaged pre-mixed foods or foods prepared in an industrial setting like a “Domino’s” pizza or “Stouffers meatloaf”. Foods with this classification were not disaggregated and Nova categorization was applied at the food item level. 
   b. *Culinary preparation:* Mixed dished item made in the home or small batch in a restaurant. All foods with this classification were disaggregated into their SII. Examples include “pizza, from homemade or restaurant” or grilled cheese when the meal location was noted as “home”.

The following Nova classification procedure was followed for all foods, excluding alcohol-containing beverages. All single ingredient items and mixed dish industrial foods were classified according to their Nova processing level. A basic flow chart for the process is shown in **Supplemental** **Figure S1B**. Nova classification was followed according to Monteiro et al. (2011), and Martinez-Steele et al. (2023) best practices as much as possible for each food and =-ingredient item (Martinez-Steele et al., 2023; Monteiro et al., 2011) (**Supplemental Figure S1C**). If a categorization was not clear based on available description of the food/ingredient from original food records, NDSR output and Nova categorization rules, the Open Food Facts database was referenced to determine if Nova categorization has already been determined for the food or ingredient (*OFF*, n.d.). For any remaining foods or ingredients unable to be categorized as described, team meetings were held until an agreement was reached. Foods that had ambiguous details, such as “unknown if salted”, a conservative assumption of greater processing was made throughout this analysis and the higher Nova level was used.

**Supplemental Tables:**

**Supplementary Table S1: Alcohol-containing beverage preference**

| **Alcohol Preference** | **AB**  **N (%)** | **CD**  **N (%)** | **HC** **N (%)** |
| --- | --- | --- | --- |
| Beer/Wine and Hard Liquor | 4 (40%) | 1 (11.11%) | 0 (0%) |
| Beer/Wine | 1 (10%) | 7 (77.88%) | 4 (33.33%) |
| Hard Liquor | 5 (50%) | 1 (11.11%) | 1 (8.33%) |
| Does not consume alcohol | 0 (0%) | 0 (0%) | 7 (58.33%) |

**Supplementary Table S2: Pairwise tests from significant variables from Table 1**

| **Measure** | **AB *vs.* CD** | | **CD *vs.* HC** | | **HC *vs.* AB** | |
| --- | --- | --- | --- | --- | --- | --- |
|  | Z score | P | Z score | P | Z score | P |
| Average Drinks per Day | -3.39 | <.001 | -3.35 | <.001 | -3.38 | .001 |
| Number Heavy Drinking Days | -3.39 | .009 | -3.58 | <.001 | -3.84 | <.001 |
| Number Heavy Drinking Years | -1.02 | .219 | -2.31 | .021 | -3.65 | <.001 |
| ADS | -2.74 | .006 | -4.03 | <.001 | -4.12 | <.001 |
| AUDIT | -2.87 | .004 | -3.82 | <.001 | -3.94 | <.001 |
| Number of years of education | 2.24 | .025 | -0.362 | .717 | 2.54 | .011 |

Post-hoc Mann Whitney tests; Abbreviation: AUDIT: Alcohol Use Disorders Identification Test and ADS: Alcohol Dependance Scale

**Supplemental Table S3: DEXA and REE measures**

|  | **AB**  **(n= 10)** | **CD**  **(n= 9)** | **HC**  **(n= 12)** | **Test Statistic** | ***ε²*** | ***P*** ^a^ |
| --- | --- | --- | --- | --- | --- | --- |
|  | **Mean (SD)** | | |  |  |  |
| Waist Circumference (cm) | 93.91 (12.77) | 90.42 (9.86) | 96.78 (11.58) | H=2.03 | 0.0 | .362 |
| Total body fat (kg) | 24.30 (10.34) | 20.95 (6.99) | 28.91 (8.93) | H=4.320 | 0.009 | .082 |
| Total trunk fat (kg) | 13.78 (6.80) | 11.53 (4.96) | 15.61 (5.75) | H=2.468 | 0.0 | .206 |
| Total lean mass (kg) | 54.59 (13.24) | 54.17 (10.73) | 53.14 (7.73) | H=0.631 | 0.0 | .592 |
| Total % Fat | 29.11% (8.86) | 26.62% (6.67) | 33.34% (6.73) | H=4.115 | .0.003 | .068 |
| Android % Fat | 36.34% (12.71) | 32.47% (10.39) | 41.12% (9.71) | H=3.080 | 0.0 | .124 |
| Gynoid % Fat | 30.48%  (9.21) | 28.38% (7.64) | 35.58% (7.72) | H=3.113 | 0.0 | .116 |
| VAT mass (kg) | 1.20 (0.71) | 1.06 (0.68) | 1.37 (1.04) | H=0.235 | 0.0 | .809 |
| Android-to-Gynoid ratio | 1.16 (0.20) | 1.14 (0.23) | 1.16 (0.25) | H=0.085 | 0.0 | .962 |
| Z-score | 0.33 (1.03) | 0.61 (1.23) | 0.51 (1.13) | H=0.265 | 0.0 | .876 |
| At expectation | 10 (100%) | 9 (100%) | 12 (100%) |  | NA | NA |
| REE kcal/day | 1646.72 (292.73) | 1637.72 (312.62) | 1602.09 (220.96) | H=0.194 | 0.0 | .911 |
| RQ (total body fuel oxidation) | 0.83 (0.03) | 0.80 (0.03) | 0.84 (0.06) | H=3.344 | 0.0 | .116 |
| Dietary intake energy^*^ /REE energy | 1.21 (0.27) | 1.48 (0.29) | 1.31 (0.21) | H=5.404 | 0.042 | .067 |

**^a^**Kruskal-Walis test for significance used for p-value. Abbreviations: VAT: visceral adipose tissue; REE: Resting Energy Expenditure, RQ: Respiratory Quotient, A/G: Android/Gynoid ratio, DEXA: Dual Energy X-ray Absorptiometry. ^*^ Dietary intake energy includes energy from alcohol.

**Supplemental Table S4: Dietary intake measures across the three groups**

| **Dietary Measure** | **AB (n= 10)** | **CD (n= 9)** | **HC (n= 12)** | **Test statistic** | ***ε²*** | **P**^a^ |
| --- | --- | --- | --- | --- | --- | --- |
| **Number of days food records collected** | 9.8 (5.3) | 9.9 (3.1) | 11.7 (3.3) | H=2.23 | 0.0 | .327 |
| Total Energy (including alcohol) (kcal) | 1879.70 (399.23) | 2420.26 (713.26) | 2069.59 (223.94) | H=5.29 | 0.387 | .071 |
| Total Energy (excluding alcohol) (kcal) | 1879.45 (399.13) | 2019.12 (518.24) | 2039.34 (238.85) | H=1.47 | 0.0 | .488 |
| Total dietary fiber (g) | 15.45 (9.66) | 19.04 (7.06) | 18.64 (5.46) | H=3.40 | 0.0 | .182 |
| Added sugars (g) | 57.15 (41.23) | 45.37 (24.43) | 54.17 (13.74) | H=0.716 | 0.0 | .699 |
| Carbohydrate | 45% (8.72) | 39.33% (7.16) | 43.94% (7.62) | H=2.92 | 0.0 | .232 |
| Protein | 18.07% (5.02) | 14.60% (1.86) | 17.43% (2.20) | H=5.99 | **0.058** | **.049** |
| Fat | 36.86% (5.84) | 31.79% (5.19) | 37.28% (5.88) | H=5.53 | 0.045 | .062 |
| Alcohol | 0.01% (0.02) | 14.41% (11.06) | 1.35% (2.31) | H=17.15 | **0.291** | **<.001** |

**^a^**Kruskal-Wallis test for significance used for p-value. Bold indicates ***p*<.05**.

**Supplementary Table S5: Dietary intake measure differences across the three groups within either non-smokers or smokers**

| **Measure** | **Smoking status** | **Test Statistic** | ***ε²*** | **P** |
| --- | --- | --- | --- | --- |
| HEI | Non-Smoking | 2.939 | 0.030 | .230 |
|  | Smoking | 2.227 | .008 | .136 |
| Nova 1 | Non-Smoking | .691 | 0.0 | .708 |
|  | Smoking | 0.000 | 0.0 | 1.00 |
| Nova 2 | Non-Smoking | 2.318 | 0.010 | .194 |
|  | Smoking | .409 | 0.0 | .522 |
| Nova 3 | Non-Smoking | 3.284 | 0.041 | .194 |
|  | Smoking | 1.136 | 0.0 | .286 |
| Nova 4 | Non-Smoking | .401 | 0.0 | .818 |
|  | Smoking | .727 | 0.0 | .394 |
| Added Sugars | Non-Smoking | 4.854 | 0.087 | .088 |
|  | Smoking | 1.136 | 0.0 | .286 |

**Supplementary Table S6: Dietary intake measure differences between smokers and non-smokers within AB or CD groups**

| **Measure** | **Group** | **Test Statistic** | ***ε²*** | **P** |
| --- | --- | --- | --- | --- |
| HEI | AB | 6.546 | 0.132 | **.011** |
|  | CD | 1.500 | .017 | .221 |
| % Energy from: |  | | | |
| Nova 1 | AB | .409 | 0.0 | .522 |
|  | CD | .060 | 0.0 | .807 |
| Nova 2 | AB | .727 | 0.0 | .394 |
|  | CD | .240 | 0.0 | .624 |
| Nova 3 | AB | 1.636 | 0.0 | .201 |
|  | CD | 1.500 | 0.0 | .221 |
| Nova 4 | AB | 1.136 | 0.0 | .286 |
|  | CD | .000 | 0.0 | 1.000 |
| Added Sugars | AB | .727 | 0.0 | .394 |
|  | CD | .240 | 0.0 | .624 |

**Supplemental Figures:**

**Supplemental Figure S1: Nova classification schema and baseline classification rules workflow**


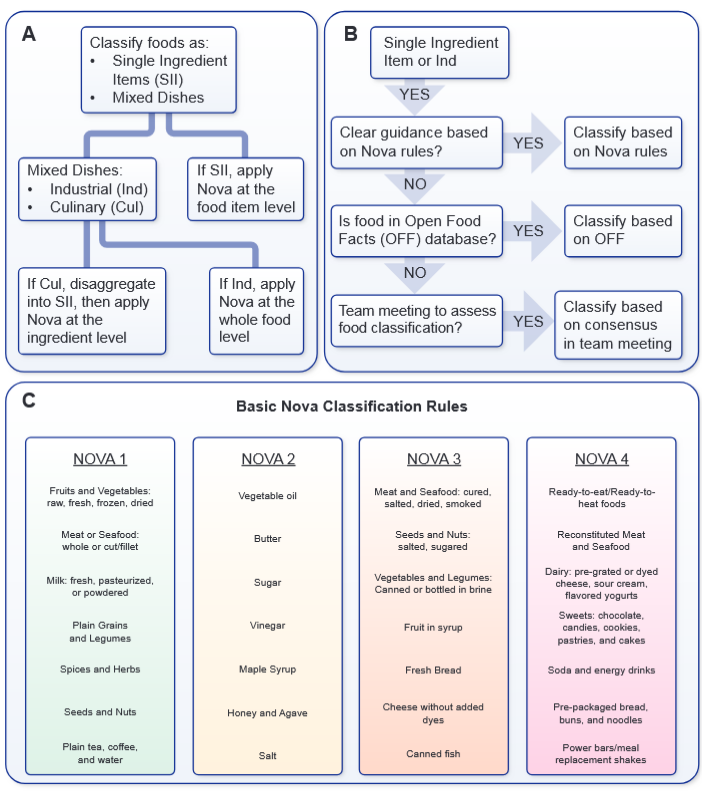


**Legend**: A) Flow chart detailing classification and disaggregation of foods. B) Flow chart detailing method of classifying food into Nova categories.  C) Chart detailing method of classifying food into Nova categories.

**Supplemental Figure S2: Days abstinent prior to enrollment in Gut-Brain microbiome study**


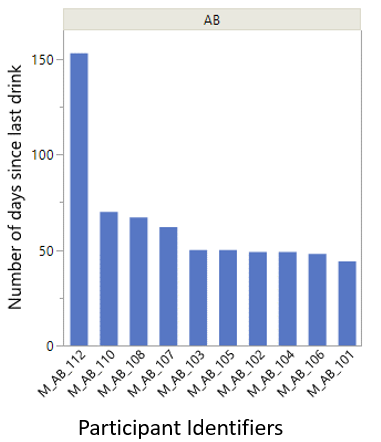

**Legend**: Histogram showing the number of days AB individuals were abstinent (y-axis) prior to enrolling in current study. X-axis indicates the participants for the AB group.

**Supplemental Figure S3: Number of food records per participant**

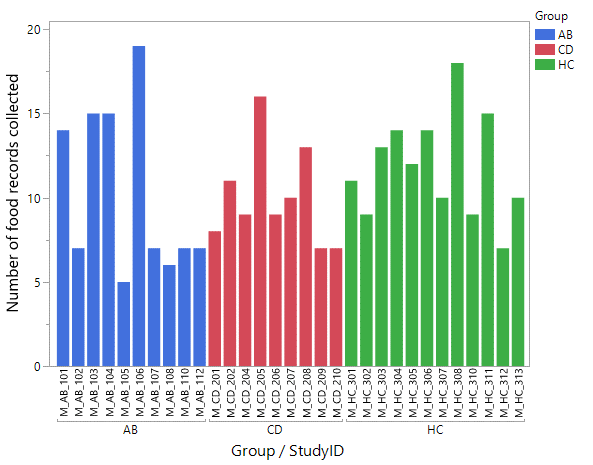


**Legend**: Total number of food records (y-axis) provided and assessed by each study participant (x-axis) across the study collection window.

**Supplemental Figure S4: Multivariate Spearman correlation coefficient heatmap between all measures within each group**


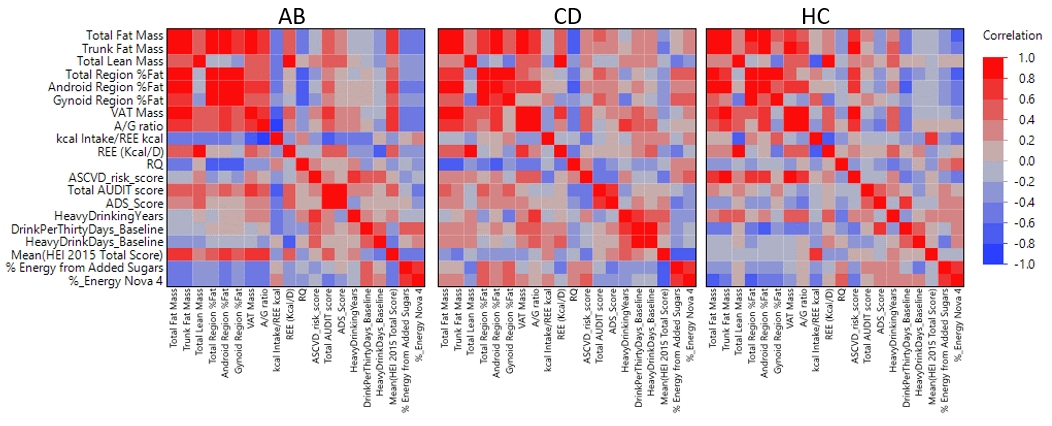


**Legend**: Multivariate correlation coefficient heatmap for all comparisons between body composition, REE, ASCVD risk, alcohol severity and use variables and dietary measures. Each heatmap shows correlations within each of the three groups. The heatmap indicates the Spearman coefficient gradient ranging from -1.0 to 1.0.

**Supplemental Figure S5: Significance correlation dot map between body composition, REE, ASCVD and drinking measures in AB and CD*
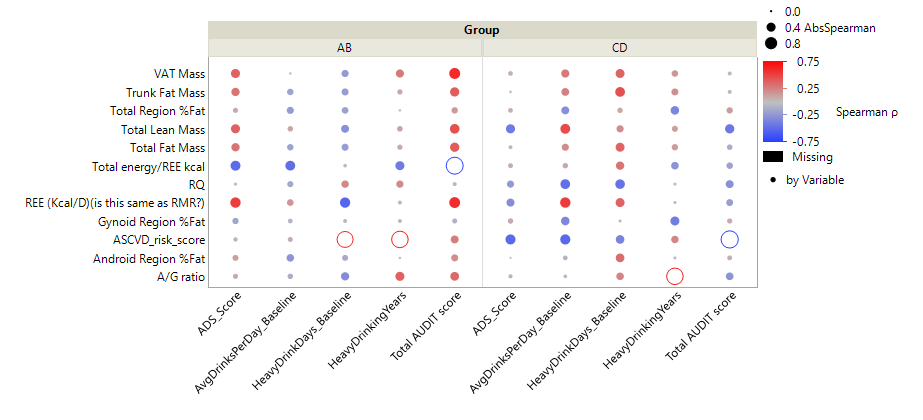
* Legend:** Spearman correlation dot map showing associations with body composition, REE, ASCVD compared with drinking severity and intake for ABs and CDs. Color of the dots indicate Spearman correlation coefficient gradient. Heatmap indicates Spearman coefficient. Size of the dot indicates absolute Spearman coefficient. Open circles indicate p<.05; no associations passed FDR<25%.

**Supplemental Figure S6: Significance correlations with Diet Quality and Body Composition within ABs**

**
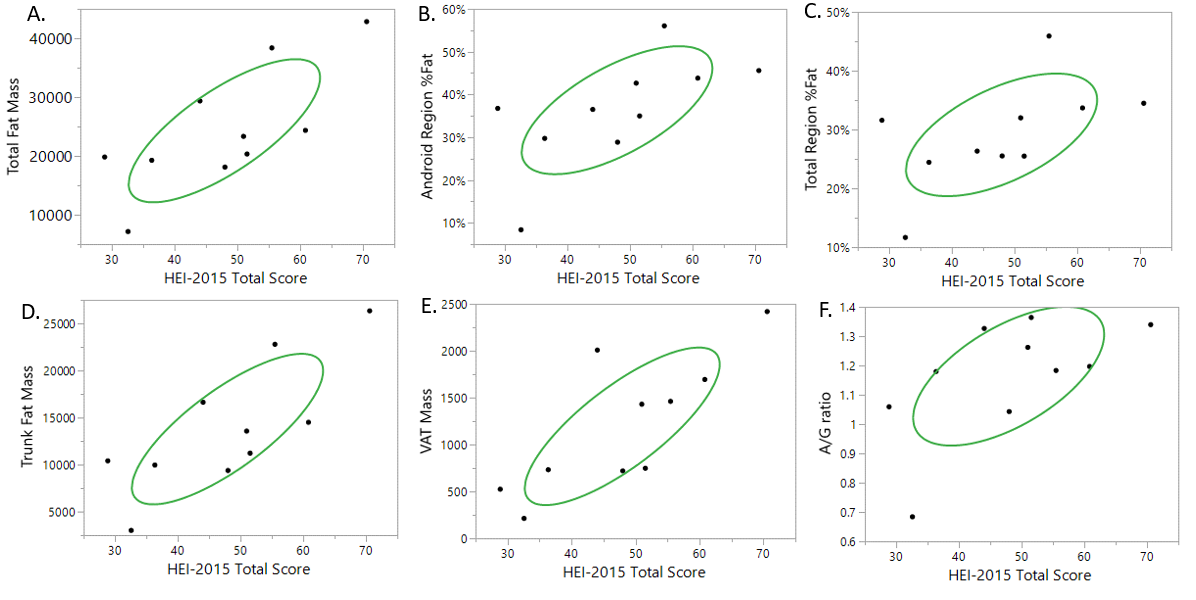
**

**Legend:** Significant associations with body composition values (y-axis) including A) total fat mass (kg), B) percent of android fat, C) percent of total fat, D) total trunk fat mass (kg), E) VAT mass, F) android to gynoid fat ratio and diet quality HEI-2015 score (x-axis) among abstinent individuals with AUD. All but A/G ratio passed FDR<25%.

**References**

Arnett, D. K., Blumenthal, R. S., Albert, M. A., Buroker, A. B., Goldberger, Z. D., Hahn, E. J., Himmelfarb, C. D., Khera, A., Lloyd-Jones, D., McEvoy, J. W., Michos, E. D., Miedema, M. D., Muñoz, D., Smith, S. C., Virani, S. S., Williams, K. A., Yeboah, J., & Ziaeian, B. (2019). 2019 ACC/AHA Guideline on the Primary Prevention of Cardiovascular Disease: Executive Summary. *Journal of the American College of Cardiology*, *74*(10), 1376–1414. https://doi.org/10.1016/j.jacc.2019.03.009

Castro, V. (2023). *CVrisk: Compute Risk Scores for Cardiovascular Diseases*. https://CRAN.R-project.org/package=CVrisk

Flanagan, B. E., Gregory, E. W., Hallisey, E. J., Heitgerd, J. L., & Lewis, B. (2011). A Social Vulnerability Index for Disaster Management. *Journal of Homeland Security and Emergency Management*, *8*(1). https://doi.org/10.2202/1547-7355.1792

Goff Jr, D. C., Lloyd-Jones, D. M., Bennett, G., Coady, S., D’agostino, R. B., Gibbons, R., Greenland, P., Lackland, D. T., Levy, D., O’donnell, C. J., & others. (2014). 2013 ACC/AHA guideline on the assessment of cardiovascular risk: A report of the American College of Cardiology/American Heart Association Task Force on Practice Guidelines. *Circulation*, *129*(25_suppl_2), S49–S73.

Martinez-Steele, E., Khandpur, N., Batis, C., Bes-Rastrollo, M., Bonaccio, M., Cediel, G., Huybrechts, I., Juul, F., Levy, R. B., Da Costa Louzada, M. L., Machado, P. P., Moubarac, J.-C., Nansel, T., Rauber, F., Srour, B., Touvier, M., & Monteiro, C. A. (2023). Best practices for applying the Nova food classification system. *Nature Food*, *4*(6), 445–448. https://doi.org/10.1038/s43016-023-00779-w

MDcalc. (n.d.). *ASCVD (Atherosclerotic Cardiovascular Disease) 2013 Risk Calculator from AHA/ACC* [Calculator]. MDcalc. Retrieved February 7, 2024, from https://www.mdcalc.com/calc/3398/ascvd-atherosclerotic-cardiovascular-disease-2013-risk-calculator-aha-acc

Monteiro, C. A., Levy, R. B., Claro, R. M., De Castro, I. R. R., & Cannon, G. (2011). Increasing consumption of ultra-processed foods and likely impact on human health: Evidence from Brazil. *Public Health Nutrition*, *14*(1), 5–13. https://doi.org/10.1017/S1368980010003241

*Open Food Facts*. (n.d.). Open Food Facts. Retrieved September 18, 2024, from https://world.openfoodfacts.org/

R Core Team. (2023). *R: A Language and Environment for Statistical Computing*. R Foundation for Statistical Computing. https://www.R-project.org/
